# Analysis of the Transient Response of a Dual-Fed RC Transmission Line

**DOI:** 10.1371/journal.pone.0116993

**Published:** 2015-02-13

**Authors:** Mohsen Dorraki, Gregory K. Cambrell, Derek Abbott

**Affiliations:** 1 Department of Electrical Engineering, Amirkabir University of Technology, Tehran, Iran; 2 Department of Electrical and Computer Systems Engineering, Faculty of Engineering, Monash University, Victoria, Australia; 3 School of Electrical and Electronic Engineering, University of Adelaide, South Australia, Australia; Glasgow University, UNITED KINGDOM

## Abstract

The transient analysis of a uniform transmission line of finite length is considered in this paper. For the first time this paper provides an analytical expression for the time-domain response of an RC transmission line, which is stimulated by a step function that is fed into both ends of the transmission line. In particular, we find an analytical expression for the step response at the center of the transmission line, in order to determine the worst-case rise time. This is of interest, for example, in large charge-coupled device (CCD) arrays, where long polysilicon lines are dual-fed in order to mitigate degradation in rise time. The analytical expressions for the RC transmission line are supported by computer-simulated lumped RC models.

## Introduction

Integrated circuits have seen significant progress—from the early developments that contained only a few transistors to modern microprocessors that can contain billions of elements. This development has been made possible by the downscaling of circuit dimensions, which results in increased speed and reduced costs.

The disadvantage of this development is that the resistance of the wires between transistors increases disproportionately when their cross-sectional dimensions are reduced. Consequently, when they are scaled down, signal propagation speed becomes reduced.

Estimating interconnection delay is an important issue for present circuits. Many authors have modeled the wires inside integrated circuits as distributed RC lines. The analysis of finite RC interconnections under a step input is widely discussed in the literature, e.g. [1, 2]. The usual method is to calculate the transfer function, then the time-domain response is extracted for an RC transmission line with a step input. Many authors have adopted different approaches to obtain a time-domain response using the Laplace transform technique [3]. Kahng and Muddu presented the time-domain response for an RC transmission line with source and load impedances [3]. Moreover, they analyzed a finite transmission line for a ramp input [4], using a method based on solving the diffusion equation and applying the method of images [3]. Rao reviewed the step response of the semi-infinite distributed RC line and focused mainly on the step response of a finite-length RC line with a capacitive load termination, which is the most common model for a wire within present-day integrated CMOS chips [5]. Additionally, Gupta and Patnaik presented an exact analysis of the output response of a distributed RC interconnect under input signals that are polynomial in time [6].

None of these previous studies used the same step input fed simultaneously into both ends of the transmission line, while considering the behavior at the center of the line. This scheme is of interest for long interconnects on chips and also for long electrodes on charged-coupled devices (CCDs). For example, in order to mitigate a slow rise time, a circular clock bus can be fed from both ends, and it becomes of interest to have an analytical expression for the transient response at the center of the bus. Similarly, in order to reduce RC degradation of clock signals along long CCD electrodes, it is possible to feed the clock to both ends of the electrodes [7]. An analytical expression that predicts the worst-case rise time at the center of the electrode is therefore a useful design equation. Another example, where such an analytical expression is potentially useful, is in the design of A and J gates for future silicon quantum computers [8].

The rest of this paper is organized as follows. After reviewing the transient response of a semi-infinite RC transmission line and an RC line of finite length with open-circuit termination, we will derive the transient response of an RC line of finite length that is fed at both ends with the same step function. In particular, we will determine the improvement of the 63% rise time thereby achieved at the center of the line. The analytical expressions will be presented in two forms: a convergent series of trigonometric and exponential functions, and a convergent series of complementary error functions. Furthermore, it will be evident that the same expressions arise by applying the method of superposition. As a final confirmation of the derived analytical expressions, the distributed RC line of finite length is modeled by several lumped RC sections, and the step response at the center of the model is computed and compared with the analytical expressions.

## Transient Response of RC Transmission Lines

### Semi-Infinite RC Transmission Line

**Figure 1:**
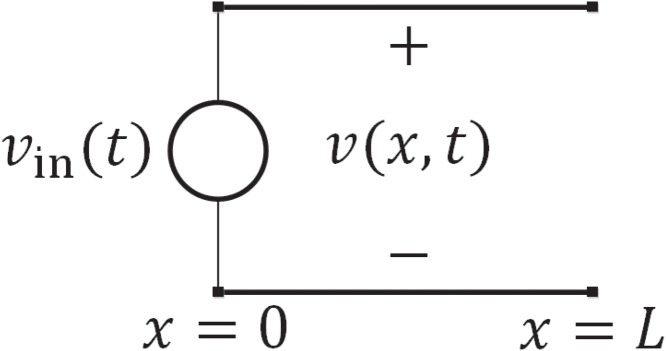
An RC transmission line.

An RC transmission line is shown in Fig. 1. This may, for example, be the model of a CCD electrode. Ignoring any propagation time due to inductance, the voltage across the line *v*(*x*, *t*) at position *x* along the line and at time *t* satisfies the well-known diffusion equation

$$\frac{\partial^{2}v}{\partial x^{2}}=rc\;\frac{\partial v}{\partial t}$$ (1)

together with appropriate boundary conditions and initial condition. Here *r* and *c* are the uniform resistance and capacitance per unit length. It is a linear partial differential equation (PDE). The corresponding current in the line *i*(*x*, *t*) satisfies the same PDE and is related to the voltage *v*(*x*, *t*) by

$$i=-\frac{1}{r}\;\frac{\partial v}{\partial x}\cdot$$ (2)

A particular integral for the diffusion equation is

$$v\left(x,t\right)=a\;\text{erfc}\left(\left(b\pm \frac{x}{L}\right)/\sqrt{\frac{4t}{rcL^{2}}}\right)+cx+d$$ (3)

where *a*, *b*, *c* and *d* are constants and erfc(⋅) is the complementary error function defined by

$$\text{erfc}\left(z\right)=1-\frac{2}{\sqrt{\pi }}\;\int_{0}^{z}\;e^{-y^{2}}\;dy\cdot$$ (4)

A characteristic length *L* has been introduced so that the dimensionless (normalized) variables

$$\chi =\frac{x}{L}$$ (5)

and

$$\tau =\frac{t}{rcL^{2}}$$ (6)

can be used throughout the analysis. The corresponding dimensionless diffusion equation is

$$\frac{\partial^{2}v}{\partial \chi^{2}}=\frac{\partial v}{\partial \tau }\cdot$$ (7)

The unit step response of a semi-infinite line extending from *x* = 0 to infinity is found by imposing the boundary conditions

$$\text{BC}1:\;v\left(0,t\right)=v_{\text{in}}\left(t\right)=u\left(t\right)\;\;\;\;\text{for}\;\text{all}\;\;\;\;t⩾0$$ (8)

$$\text{BC}2:\;i\left(\infty ,t\right)=0\;\;\text{or}\;\;\frac{\partial v}{\partial x}\left(\infty ,t\right)=0\;\;\;\;\text{for}\;\text{all}\;\;\;\;t⩾0$$ (9)

where *u*(*t*) is the unit step function, and the initial condition

IC:v(x,0)=0forall0<x<∞. (10)

Therefore *a* = 1, and *b*, *c* and *d* must be zero, and so the unit step response of the semi-infinite line is

$$v\left(x,t\right)=\text{erfc}\left(\left(\frac{x}{L}\right)/\sqrt{\frac{4t}{rcL^{2}}}\right)$$ (11)

or

$$v\left(\chi ,\tau \right)=\text{erfc}\left(\chi /\sqrt{4\tau }\right).$$ (12)

### Finite RC Transmission Line with Open-Circuit Termination

Consider an RC transmission line of finite length *L* with open-circuit termination, as shown in Fig. 1, fed with a unit step function of voltage. Rao has solved this case by using the Laplace transform technique [5]. For the Laplace transform of the voltage step response *v*(*x*, *t*) he obtains

$$L{\left\{v(x,t)\right\}}_{s}^{t}=\int_{0}^{\infty }v\left(x,t\right)e^{-st}\;dt=V\left(x,s\right)=\frac{1}{s}\frac{\text{cosh}\left(\left(1-\frac{x}{L}\right)\sqrt{rcL^{2}s}\right)}{\cosh(\sqrt{rcL^{2}s})}$$ (13)

for 0 ≤ *x* ≤ *L*. By directly inverting this Laplace transform Rao obtains the following infinite series for the unit step response:

$$v\left(x,t\right)=1-\frac{2}{\pi }\sum_{n=1}^{\infty }\frac{1}{\left(n-\frac{1}{2}\right)}\left[\sin\left(\left(n-\frac{1}{2}\right)\pi \left(\frac{x}{L}\right)\right)\right]e^{-{\left((n-\frac{1}{2})\pi \right)}^{2}t/\left(rcL^{2}\right)}.$$ (14)

Then, by using the expansion

$$\frac{1}{\cosh(y)}=\frac{2e^{-y}}{1+e^{-2y}}=2\sum_{n=0}^{\infty }{\left(-1\right)}^{n}e^{-(2n+1)y}$$ (15)

so that

$$V\left(x,s\right)=\frac{1}{s}\sum_{n=0}^{\infty }{\left(-1\right)}^{n}\left\{e^{-\left(2n+\frac{x}{L}\right)\sqrt{rcL^{2}s}}+e^{-\left(2n+2-\frac{x}{L}\right)\sqrt{rcL^{2}s}}\right\}$$ (16)

he performs an alternative inversion of the Laplace transform (13) and obtains another, but equivalent, infinite series for the unit step response:

$$v\left(x,t\right)=\sum_{n=0}^{\infty }{\left(-1\right)}^{n}\left\{\text{erfc}\left(\left(2n+\frac{x}{L}\right)/\sqrt{\frac{4t}{rcL^{2}}}\right)+\text{erfc}\left(\left(2n+2-\frac{x}{L}\right)/\sqrt{\frac{4t}{rcL^{2}}}\right)\right\}.$$ (17)

For the purpose of comparison, the following practical example will be considered here and later in the paper. Let *r* = 1 Ω/m, *c* = 1×10^-6^ F/m and *L* = 1×10^-3^ m. The unit step response at the center of this line will be of particular interest. Here, *x* = 0.5×10^-3^ m and *χ* = *x*/*L* = 0.5. When evaluated at this position, the unit step response reaches (1–1/*e*) or 0.63212 of its final value of 1.0 in the time *t* = 0.36283×10^-12^ s, that is, when *τ* = *t*/(*rcL* ^2^) = 0.36283. This is called the 63% rise time.

For interest, if this practical line were extended to infinity (becoming a semi-infinite line), the 63% rise time at the same position would increase to time *t* = 0.54538×10^-12^ s, that is, to *τ* = *t*/(*rcL* ^2^) = 0.54538. The increased rise time is due to the need to charge the extra capacitance beyond *x* = *L*.

On the other hand, if this practical line is fed with the same step function of voltage into both ends of the line, charging of the capacitance within the finite line is potentially faster than when leaving the far end open.

### Analysis of Dual-Fed RC Transmission Line

**Figure 2:**
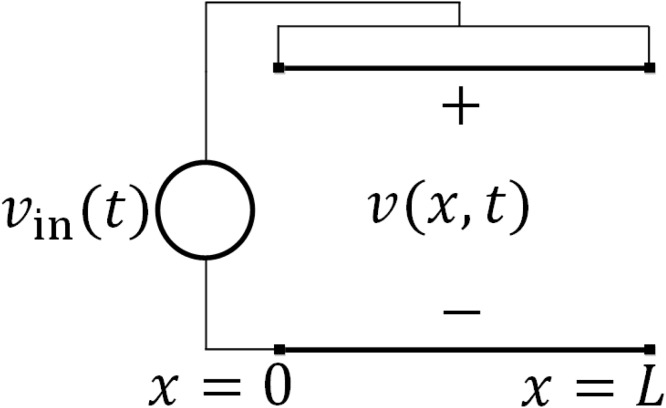
The RC transmission line is identical to that in Fig. 1, except that both ends are fed with the same step function *v_in_*(*t*). The response of interest, *v*(*x*; *t*), is the voltage at the center of the line. The voltage at that position will possess the worst-case rise time.

Consider an RC transmission line of finite length *L* fed with the same step function of voltage at both ends, as shown in Fig. 2. The boundary and initial conditions now become

$$\text{BC}1:\;v\left(0,t\right)=u_{1}\cdot u\left(t\right)\;\;\;\;\text{for}\;\text{all}\;\;\;\;t⩾0,$$ (18)

$$\text{BC}2:\;v\left(L,t\right)=u_{2}\cdot u\left(t\right)\;\;\;\;\text{for}\;\text{all}\;\;\;\;t⩾0,$$ (19)

IC:v(x,0)=0forall0<x<L. (20)

For generality, individual step amplitudes *u*_1_ and *u*_2_ have been introduced at each end of the RC line. They can both be set to unity at the end of the analysis.

Taking the Laplace transform of the diffusion Equation (1) and applying the initial condition (20) gives the wave equation

$$\frac{\partial^{2}}{\partial x^{2}}V\left(x,s\right)=rc{\left[v\left(x,t\right)e^{-st}\right]}_{t=0}^{t=\infty }+rcs\int_{0}^{\infty }v\left(x,t\right)e^{-st}dt=rcsV\left(x,s\right).$$ (21)

Its general solution in the Laplace transform domain is

$$V\left(x,s\right)=A\left(s\right)\;e^{x\sqrt{rcs}}+B\left(s\right)\;e^{-x\sqrt{rcs}}$$ (22)

where *A*(*s*) and *B*(*s*) do not depend on *x* and must be determined from the boundary conditions (18) and (19). Since the Laplace transform of the unit step function is 1/*s*, BC1 and BC2 become

$$V\left(0,s\right)=A\left(s\right)+B\left(s\right)=\frac{u_{1}}{s},$$ (23)

$$V\left(L,s\right)=A\left(s\right)\;e^{L\sqrt{rcs}}+B\left(s\right)\;e^{-L\sqrt{rcs}}=\frac{u_{2}}{s}\cdot$$ (24)

Thus

$$A\left(s\right)=\frac{-u_{1}e^{-L\sqrt{rcs}}+u_{2}}{2s\;\sinh(L\sqrt{rcs})},$$ (25)

$$B\left(s\right)=\frac{u_{1}e^{L\sqrt{rcs}}-u_{2}}{2s\;\sinh(L\sqrt{rcs})}$$ (26)

so that

$$V\left(x,s\right)=\frac{1}{s}.\frac{u_{1}\;\sinh\left(\left(1-\frac{x}{L}\right)\sqrt{rcL^{2}s}\right)+u_{2}\;\sinh\left(\left(\frac{x}{L}\right)\sqrt{rcL^{2}s}\right)}{\sinh(\sqrt{rcL^{2}s})}=u_{1}F\left(L-x,s\right)+u_{2}F\left(x,s\right)$$ (27)

where

$$F\left(x,s\right)=\frac{1}{s}\cdot \frac{\sinh\left(\left(\frac{x}{L}\right)\sqrt{rcL^{2}s}\right)}{\sinh\left(\sqrt{rcL^{2}s}\right)}\cdot$$ (28)

The inverse Laplace transform of *F*(*x*, *s*) is

$$f\left(x,t\right)=L^{-1}{\left\{F(x,s)\right\}}_{t}^{s}=\frac{1}{2\pi j}\int_{\alpha -j\infty }^{\alpha +j\infty }F\left(x,s\right)e^{ts}ds=\frac{1}{2\pi j}\int_{\alpha -j\infty }^{\alpha +j\infty }\frac{1}{s}\cdot \frac{\sinh\left(\left(\frac{x}{L}\right)\sqrt{rcL^{2}s}\right)}{\sinh\left(\sqrt{rcL^{2}s}\right)}\;e^{ts}ds.$$ (29)

Though sinh$\sqrt{z}$ is not analytic, the ratio of two such functions is lacking any double-valuedness and so there is no need for a cut along the negative-real axis. Note that this ratio has a line of first-order poles along this axis. For *t* < 0, the contour of the integration may be closed to the right, the integral at infinity goes to zero, and the value of the contour integral is zero. For *t* > 0, this is not permissible, the integral becoming exponentially large due to the *e* ^*ts*^ factor. However, the contour may be closed to the left, thus enclosing the line of poles. The value of the new integral at infinity is still zero, and the contour integral that we are interested in takes on the value of the sum of the residues. The simple poles are located at

$$s_{n}=-\frac{{\left(n\pi \right)}^{2}}{rcL^{2}}$$ (30)

for each integer *n* ≥ 1. Therefore,

$$f\left(x,t\right)=\frac{x}{L}+\sum_{n=1}^{\infty }\frac{2}{jn\pi }\frac{\sinh\left(jn\pi x/L\right)e^{-{\left(n\pi \right)}^{2}t/\left(rcL^{2}\right)}}{\cosh(jn\pi )}$$ (31)

so that

$$f\left(x,t\right)=\frac{x}{L}+\frac{2}{\pi }\sum_{n=1}^{\infty }\frac{{\left(-1\right)}^{n}}{n}\sin\left(n\pi x/L\right)e^{-{\left(n\pi \right)}^{2}t/\left(rcL^{2}\right)}.$$ (32)

Hence the general step response is

$$v\left(x,t\right)=u_{1}\left(1-\frac{x}{L}\right)+u_{2}\left(\frac{x}{L}\right)+\frac{2}{\pi }\sum_{n=1}^{\infty }\frac{{\left(-1\right)}^{n}}{n}\left[u_{1}\;\sin\left(n\pi \left(1-\frac{x}{L}\right)\right)+u_{2}\;\sin\left(n\pi \left(\frac{x}{L}\right)\right)\right]e^{-{\left(n\pi \right)}^{2}t/\left(rcL^{2}\right)}.$$ (33)

Finally, by setting *u*_1_ = 1 and *u*_2_ = 1, the unit step response of the dual-fed RC line is

$$v\left(x,t\right)=1+\frac{2}{\pi }\sum_{n=1}^{\infty }\frac{{\left(-1\right)}^{n}}{n}\left[\sin\left(n\pi \left(1-\frac{x}{L}\right)\right)+\sin\left(n\pi \left(\frac{x}{L}\right)\right)\right]e^{-{\left(n\pi \right)}^{2}t/\left(rcL^{2}\right)}.$$ (34)

At the center of the line where *χ* = *x*/*L* = 0.5, the unit step response of the dual fed RC line is

$$v\left(\frac{L}{2},t\right)=1-\frac{4}{\pi }\sum_{n=0}^{\infty }\frac{{\left(-1\right)}^{n}}{\left(2n+1\right)}e^{-{\left((2n+1)\pi \right)}^{2}t/\left(rcL^{2}\right)}$$ (35)

or

$$v\left(0.5,\tau \right)=1-\frac{4}{\pi }\sum_{n=0}^{\infty }\frac{{\left(-1\right)}^{n}}{\left(2n+1\right)}e^{-{\left((2n+1)\pi \right)}^{2}\tau }.$$ (36)

Again let *r* = 1 Ω/m, *c* = 1×10^-6^ F/m and *L* = 1×10^-3^ m. The unit step response at the center of this dual-fed RC line reaches (1–1/*e*) or 0.63212 of its final value of 1.0 in the time *t* = 0.125795×10^-12^ s, that is, when *τ* = *t*/(*rcL* ^2^) = 0.125795.

Compared to the case when this line is left open at the far end, the 63% rise time at the center has been reduced by a factor of 2.8843, which is a significant improvement.

### Alternative Analysis of Dual-Fed RC Transmission Line

Following a method similar to that presented by Rao in [5], and using the expansion

$$\frac{1}{\sinh(y)}=\frac{2e^{-y}}{1-e^{-2y}}=2\sum_{n=0}^{\infty }e^{-(2n+1)y}$$ (37)

the Laplace transform (28) can be expanded as

$$F\left(x,s\right)=\frac{1}{s}\sum_{n=0}^{\infty }\left\{e^{-\left(2n+1-\frac{x}{L}\right)\sqrt{rcL^{2}s}}-e^{-\left(2n+1+\frac{x}{L}\right)\sqrt{rcL^{2}s}}\right\}.$$ (38)

Then another, but equivalent, infinite series for the general step response of the dual-fed RC line is

$$\begin{array}{cc} v\left(x,t\right)=\sum_{n=0}^{\infty } & \left\{\;u_{1}\left[\text{erfc}\left(\left(2n+\frac{x}{L}\right)/\sqrt{\frac{4t}{rcL^{2}}}\right)-\text{erfc}\left(\left(2n+2-\frac{x}{L}\right)/\sqrt{\frac{4t}{rcL^{2}}}\right)\right]+u_{2}\left[\text{erfc}\left(\left(2n+1-\frac{x}{L}\right)/\sqrt{\frac{4t}{rcL^{2}}}\right)-\text{erfc}\left(\left(2n+1+\frac{x}{L}\right)/\sqrt{\frac{4t}{rcL^{2}}}\right)\right]\right\}. \end{array}$$ (39)

The unit step response is obtained by setting *u*_1_ = 1 and *u*_2_ = 1. At the center of the line where *χ* = *x*/*L* = 0.5, the unit step response of the dual-fed RC line is

$$v\left(\frac{L}{2},t\right)=2\sum_{n=0}^{\infty }\left\{\text{erfc}\left(\left(2n+\frac{1}{2}\right)/\sqrt{\frac{4t}{rcL^{2}}}\right)-\text{erfc}\left(\left(2n+\frac{3}{2}\right)/\sqrt{\frac{4t}{rcL^{2}}}\right)\right\}$$ (40)

or

$$v\left(0.5,\tau \right)=2\sum_{n=0}^{\infty }\left\{\text{erfc}\left(\left(2n+\frac{1}{2}\right)/\sqrt{4\tau }\right)-\text{erfc}\left(\left(2n+\frac{3}{2}\right)/\sqrt{4\tau }\right)\right\}.$$ (41)

This series has been found to converge faster than the series in Equation (36) for small values of *τ* but otherwise gives identical numerical values.

Some analytical results are shown in Table 1.

**Table 1:** Analytical results for the unit step response at the center of a dual-fed RC line.

*τ*	**0.01**	**0.02**	**0.03**	**0.05**	**0.07**	**0.10**	**0.12**	**0.13**	**0.15**	**0.20**	**0.50**
*v*(0.5, *τ*) (volt)	0.001	0.025	0.082	0.228	0.363	0.526	0.610	0.647	0.710	0.823	0.991

### Analysis of Dual-Fed RC Transmission Line using Superposition

For any bilateral linear electrical network, lumped or distributed, the method of superposition is valid. In particular, the voltage at any point in an RC transmission line that is fed by two independent sources is the algebraic sum of the voltages caused by each independent source acting alone, where the other source is reduced to zero and replaced by its internal impedance. Thus, an ideal voltage source becomes a short-circuit when reduced to zero.

The problem of the dual-fed RC transmission line can now be solved by first finding the step response within the line when it is fed by a unit step voltage source at one end having terminated the other end of the line with a short-circuit. Because of the symmetry of the uniform RC transmission line, the step response when the unit step voltage source and the short-circuit load are swapped can be found by simply changing the position variable *x* to *L*—*x*, where *L* is the length of the line. By applying superposition, the step response of the dual-fed line is the algebraic sum of the step responses of the line when fed from just one end and terminated at the other end with a short-circuit.

Because of the symmetry of the uniform line the step response at the center will be the same whether it is fed from the left end and short-circuited at the right end or fed from the right end and short-circuited at the left end. Hence, the step response at the center of a dual-fed line is the same as that from a singly-fed line, terminated in a short-circuit, but fed with double the amplitude.

The step response of a forward-fed finite RC transmission line with short-circuit termination at *x* = *L* can obtained by setting *u*_2_ = 0 in either Equation (33) or Equation (39). Similarly the step response of a backward-fed finite RC transmission line with short-circuit termination at *x* = 0 can obtained by setting *u*_1_ = 0 in either Equation (33) or Equation (39). By superposition, the general step response of a dual-fed RC line is the algebraic sum of these two individual step responses. But that is exactly what appears in both Equations (33) and (39).

Furthermore, it is easily shown that the step response at the center of a dual-fed RC line is simply halved in amplitude when the source at either end is reduced to zero and replaced by a short-circuit. By linearity, that halved-response can be restored by doubling the amplitude of the remaining source. Shorting the far end of an RC line maximizes the current through the bulk of the transmission line, but at the same time causes the greatest voltage drop towards the far end. It follows that terminating the far end of the line with any finite value of resistance will improve the rise time within the RC line compared to when that far end is left open. The source amplitude can be increased to compensate for the extra voltage drop towards the far end.

## Transient Response of Lumped RC Models

**Figure 3:**
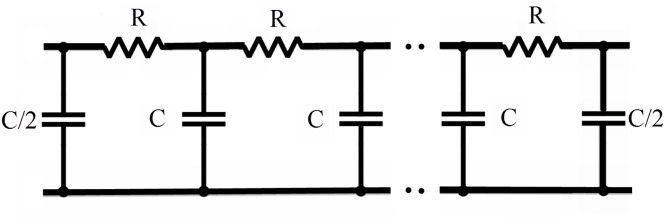
The lumped RC model.

A lumped approximation is used to model the distributed RC line as lumped resistors and lumped capacitors, as shown in Fig. 3. As the transmission line is uniform, the lumped model consists of several identical π sections in cascade.

**Figure 4:**
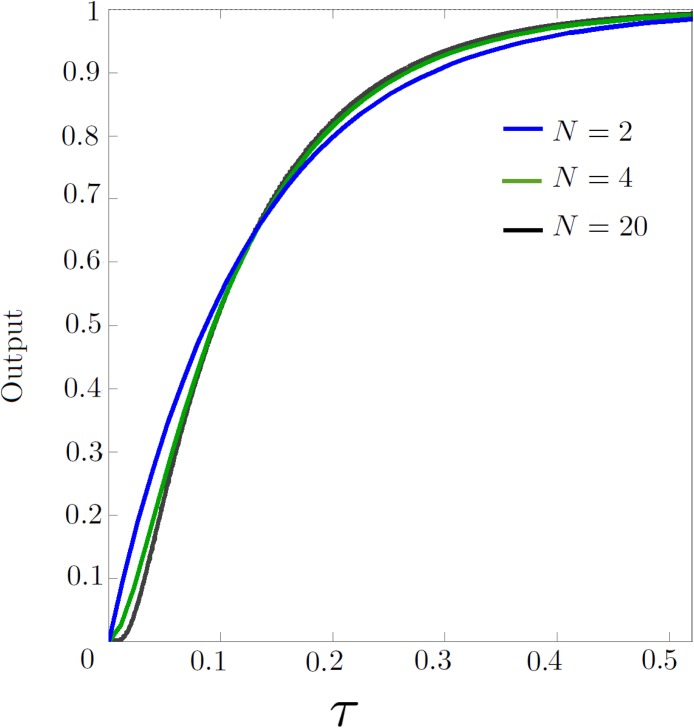
The voltage at the center of the dual-fed line, simulated with *N* = 2, 4 and 20 sections.

A model with two π sections in cascade is the simplest approximation to the transmission line. A better approximation has four sections, wherein the transmission line is split into two halves, and each half is approximated by two π sections in cascade. The approximation continues to improve as the line is broken into smaller lengths, each piece being represented by a π section. Table 2 lists the computed results for the unit step response at the center of the dual-fed lumped model with *N* sections where *N* = 2,4,10 and 20. The example values of *r* = 1 Ω/m, *c* = 1×10^-6^ F/m and *L* = 1×10^-3^ m were used in the computations, but the results are shown for selected values of dimensionless (normalized) time *τ* = *t*/(*rcL* ^2^). The results for *N* = 2,4 and 20 are plotted in Fig. 4.

**Table 2:** Analytical and lumped model results for the unit step response at the center of a dual-fed RC line.

*τ*	analytical	*N* = 2	*N* = 4	*N* = 10	*N* = 20
0.01	0.001	0.077	0.021	0.004	0.002
0.02	0.025	0.148	0.069	0.034	0.027
0.03	0.082	0.213	0.129	0.091	0.085
0.05	0.228	0.330	0.258	0.232	0.229
0.07	0.363	0.429	0.378	0.365	0.363
0.10	0.526	0.551	0.528	0.526	0.526
0.12	0.610	0.617	0.608	0.610	0.610
0.13	0.647	0.647	0.643	0.646	0.647
0.15	0.710	0.699	0.704	0.709	0.710
0.20	0.823	0.798	0.815	0.822	0.823
0.50	0.991	0.982	0.989	0.991	0.991

It is clear from these plots that the lumped approximation improves as the number of sections increases. In practice, for *N* = 20, the step response closely follows the analytical result.

For reference, the step responses of the first two models, *N* = 2 and *N* = 4, have explicit expressions:

$$v_{2}\left(0.5,\tau \right)=1-e^{-8\tau }$$ (42)

and

$$v_{4}\left(0.5,\tau \right)=1-\frac{\sqrt{2}+1}{2}e^{-(32-16\sqrt{2})\tau }+\frac{\sqrt{2}-1}{2}e^{-(32+16\sqrt{2})\tau }$$ (43)

The latter is the step response of an overdamped second-order circuit with a natural frequency of $16\sqrt{2}/(rcL^{2})$ rad/s and a Q-factor of $1/(2\sqrt{2})=0.354$.

## Conclusion

This paper derives analytical expressions for the transient time response of an RC transmission line under a step excitation fed into both ends of the line. Of particular interest is the response at the center of the line, where the rise time is the longest. Two forms of the analytical expressions are obtained by inverting the Laplace transform in two ways. Both forms are infinite series but one of them converges faster for small values of time. Furthermore, the expressions were presented in a form consistent with the method of superposition.

It is found that the 63% rise time at the center is reduced by a factor of 2.8843 when the RC line is dual-fed compared to the case when it is left open at the far end.

While closed-form analytical expressions now exist for the step response at the center of a dual-fed distributed RC line, lumped models with different numbers of sections were used to approximate the line. We demonstrated that the response errors from the lumped approximations reduce significantly as the number of sections in the π model is increased, 20 sections being enough in practice to closely predict the step response of the distributed line.
