# Correction: Analysis of the Transient Response of a Dual-Fed RC Transmission Line

**DOI:** 10.1371/journal.pone.0123497

**Published:** 2015-03-30

**Authors:** The PLOS ONE Staff

In the Alternative Analysis of Dual-Fed RC Transmission Line sub-section of the Transient Response of RC Transmission Lines section there is an error in the 39^th^ equation in the PDF version. The equation is incorrectly formatted. Please view the complete, correct equation here:

$$\begin{array}{l} v(x,t)=\sum_{n=0}^{\infty }\{u_{1}\left[\text{erfc}\left(\left(2n+\frac{x}{L}\right)/\sqrt{\frac{4t}{rcL^{2}}}\right)-\text{erfc}\left(\left(2n+2-\frac{x}{L}\right)/\sqrt{\frac{4t}{rcL^{2}}}\right)\right]\\ \;+u_{2}\left[\text{erfc}\left(\left(2n+1-\frac{x}{L}\right)/\sqrt{\frac{4t}{rcL^{2}}}\right)-\text{erfc}\left(\left(2n+1+\frac{x}{L}\right)/\sqrt{\frac{4t}{rcL^{2}}}\right)\right]\}. \end{array}$$ (39)
